# Behavioral correlates of activity of optogenetically identified locus coeruleus noradrenergic neurons in rats performing T-maze tasks

**DOI:** 10.1038/s41598-018-37227-w

**Published:** 2019-02-04

**Authors:** Liyang Xiang, Antoine Harel, HongYing Gao, Anthony E. Pickering, Susan J. Sara, Sidney I. Wiener

**Affiliations:** 1 Center for Interdisciplinary Research in Biology (CIRB), College de France, CNRS, INSERM, PSL Research University, Paris, France; 2 School of Physiology, Pharmacology & Neuroscience, University of Bristol, Medical Sciences Building, University Walk, Bristol, BS8 1TD UK; 3 Department of Child and Adolescent Psychiatry, New York University Medical School, New York, NY USA

## Abstract

The nucleusLocus Coeruleus (LC) is the major source of forebrain norepinephrine. LC is implicated in arousal, response to novelty, and cognitive functions, including decision-making and behavioral flexibility. One hypothesis is that LC activation promotes rapid shifts in cortical attentional networks following changes in environmental contingencies. Recent recordings further suggest LC is critical for mobilizing resources to deal with challenging situations. In the present study optogenetically identified LC neuronal activity was recorded in rats in a self-paced T-maze. Rats were trained on visual discrimination; then place-reward contingencies were instated. In the session where the animal shifted tasks the first time, the LC firing rate after visual cue onset increased significantly, even as the animal adhered to the previous rule. Firing rate also increased prior to crossing photodetectors that controlled stimulus onset and offset, and this was positively correlated with accelerations, consistent with a role in mobilizing effort. The results contribute to the growing evidence that the noradrenergic LC is essential for behavioral adaptation by promoting cognitive flexibility and mobilizing effort in face of changing environmental contingencies.

## Introduction

The importance of the noradrenergic nucleus Locus Coeruleus (LC) in cognitive processes is well-acknowledged, yet little understood. Several different approaches have been used to elucidate the role of noradrenaline in learning and memory including lesions, pharmacology, electrical stimulation and unit recordings. The results inspired theories of LC function emphasizing roles in arousal, attention, memory processing and reconsolidation, decision making, and cognitive flexibility^1–5^. Electrophysiological recordings of these neurons have provided convincing evidence concerning the role of this system in cognition, but there is clearly a dearth of such studies in behaving rodents due to the difficulty of recording from such a small nucleus (1500 neurons in rats) located deep in the pontine region of the brain.

Early recordings from the LC in rats supported a role in vigilance^6^. These neurons respond with a phasic burst to novel sensory stimuli of all modalities, but habituate rapidly^7^ unless the stimulus predicts a reward^8^. They show remarkable plasticity in a formal conditioning protocol, in that they are activated by any change in the reward contingency during conditioning, i.e., extinction or reversal^8^. Engagement of the LC in response to changes in environmental contingencies is rapid^9^, sometimes occurring after a single informative trial^8^. A simultaneous recording study showed that this rapid habituation occurs in LC well before the prefrontal cortex (PFC), and well before behavioral adaptation to the new contingencies^10^. Moreover, within trials, the response latency was much shorter in LC than in PFC^10^, indicating that the LC response was not mediated through the PFC, as had been hypothesized. These results, and others from pharmacological studies suggesting that NE facilitates performance in reversal and extra-dimensional shift tasks^11,12^, led to a theory of function of the LC-NE system termed ‘network reset’^13^.

Functional Magnetic Resonance Imaging (fMRI) studies in nonhuman primates and humans confirm the engagement of the LC region in tasks requiring reorienting and rapid cognitive shifts^14^ and show co-activation of frontal cortex and LC region during ‘challenge driven attention’^15^. Recent electrophysiological recordings in monkeys have supported the notion that LC is activated by stimuli signaling forthcoming environmental challenges requiring mobilization of effort to perform the task^16^. The temporal precision afforded by electrophysiological recordings also permitted correlation of phasic LC neuronal activity with measures of autonomic arousal. This led to the proposal that LC activation is part of a general orienting response facilitating adaptation to any environmental challenge^16^ (see also ref.^4^).

**Figure 1:**
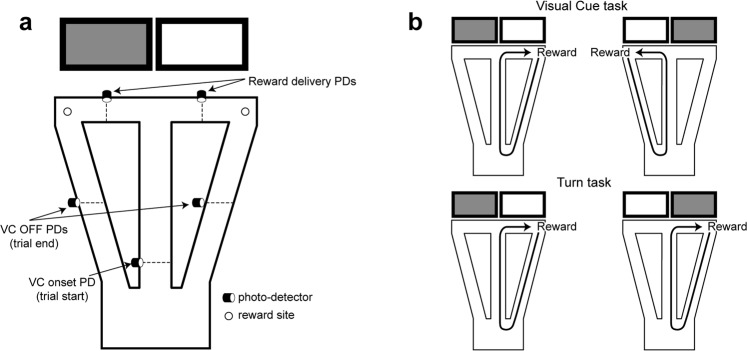
The behavioral task. (**a**) Schematic of the automated T-maze with return arms. When the rat crosses the visual cue (VC) onset photodetector (PD), this triggers one of the two screens behind the reward arms to be lit in pseudo-random sequence. Crossing the appropriate reward arm photodetector triggers the delivery of a drop of liquid reward at the corresponding reward site. Crossing the return arm (‘VC OFF’) photodetector triggers the lit screen to be turned off. (**b**) The two task rules. Under the ‘VC rule’, rats were only rewarded by choosing the reward arm in front of the lit screen regardless of whether it was to the left or the right. Under the ‘Turn rule’, rats were only rewarded on the same (non-preferred) side, regardless of which screen was lit.

The present study was designed to determine the events that elicit responses in LC neurons in a task involving reward-cued task contingency shifts and mobilization of effort. The animals moved freely, performing a self-paced automated task where their movements across photodetector beams triggered the trial onset, switched cues on and off, and then triggered reward, when appropriate (Fig. 1). Reward contingencies were shifted within sessions in well-trained rats in order to record LC responses to this challenge along with behavioral adaptation to the shift. LC neurons responded only to the first such contingency shift. Accelerations, reflecting task-related effort, were significantly correlated with increases in LC activity. Overall the results support the notion that the noradrenergic system is critical for mobilizing resources and preparing the appropriate response to face challenging situations^4,16^.

## Results

### Behavior

The 30 recording sessions included 44 to 100 trials. Post-surgery reacquisition of the visual cue task required from zero to five trials. The rule was switched when the rats reached a criterion performance of at least 18 correct out of the last 20 trials. This stringent criterion was used to achieve a relatively stable long baseline recording and assure that the relevant cues dominated the attentional set of the rat. Rule shifts were imposed once in 16 sessions, twice in two sessions and three times in one session (see Methods section).

### Identification of LC neurons

**Figure 2:**
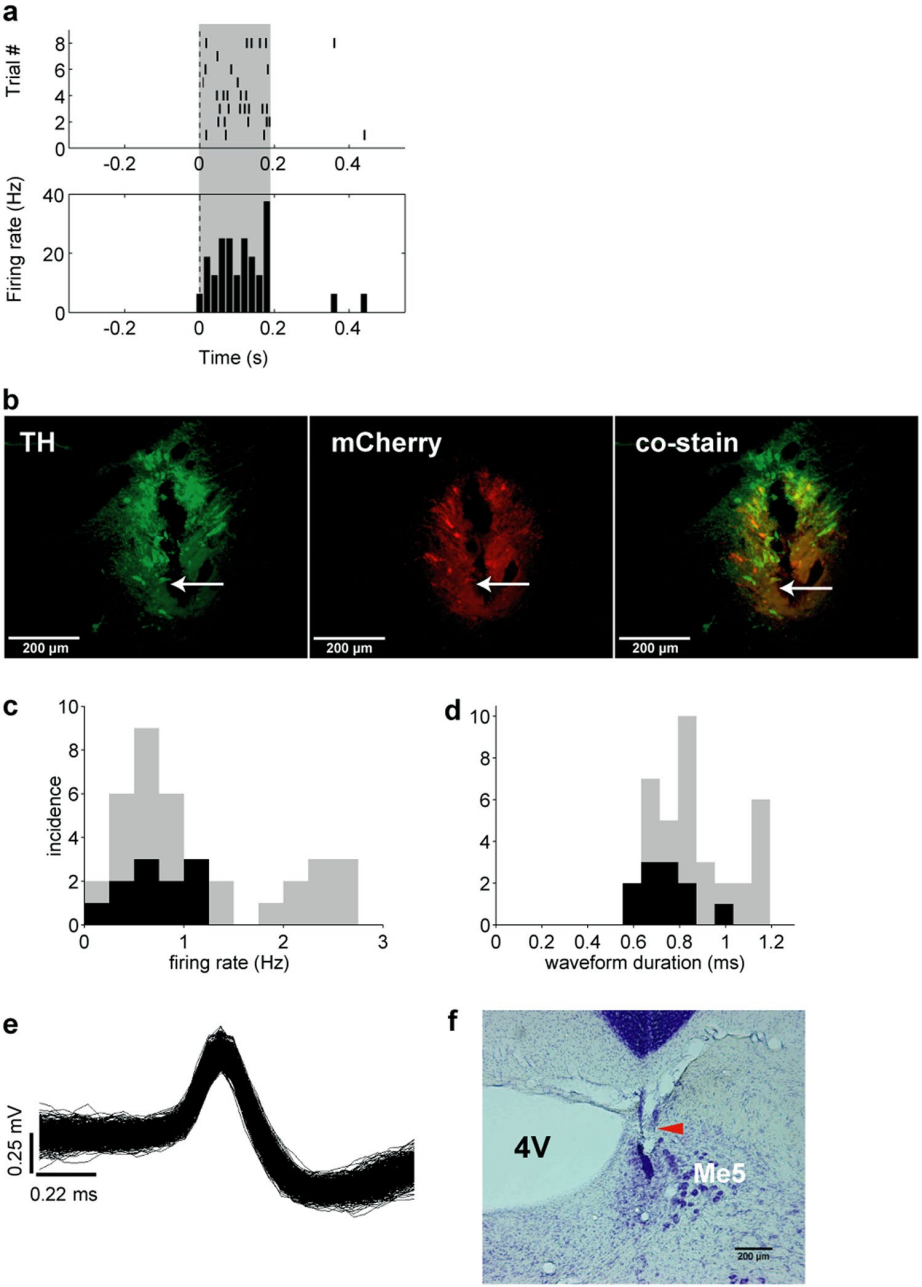
Characterization of unit recordings. (**a**) Optical responses to 2 Hz, 100 ms, 10 mW pulse train laser stimulation in a unit in the virus injected rat (R328). (**b**) Immuno-histochemical preparations showing electrode track and neurons transduced with CAV2-PRS-ChR2-mCherry. (**c**) Distribution of average firing rates of units recorded. Optogenetically identified noradrenergic units (rat R328) are shaded black. (**d**) Distribution of spike widths of units recorded. Optogenetically identified noradrenergic units are shaded black. (**e**) Left) Overdrawn waveforms of a single unit recorded in LC in a behaving rat. (**f**) Nissl stained section showing the placement of the LC electrode in one of the recorded rats. Red arrow indicates the electrode tip position. TH, Tyrosine Hydroxylase; 4V, fourth ventricle; Me5, mesencephalic trigeminal nucleus.

In one rat (R328) the noradrenergic nature of the recordings was verified optogenetically. Before each recording session in this rat, LC single or multi-unit activity was identified by responses to laser light stimulation (Fig. 2a). Immunochemistry histological results confirmed the selective expression of ChR2 in LC NE neurons as well as the location of the electrode track (Fig. 2b). Firing rates and waveform durations of the identified neurons (Fig. 2c,d, black bars) resemble those described previously^10,17^. The characteristics of all LC units of the other rats resembled the optogenetically identified ones of this rat, as did the behavioral correlates presented below. The distribution of mean firing rates per session ranged from 0.1 to 2.8 Hz (Fig. 2c; n = 37, for 4 rats, including rat R332 which had no VC task pre-training). The mean duration of spikes was 0.86 ± 0.03 ms (average of session averages; Fig. 2d). Examples of waveforms and of an LC electrode position are shown in Fig. 2e,f.

### Contingency shifts and changes in LC task-related responses

**Figure 3:**
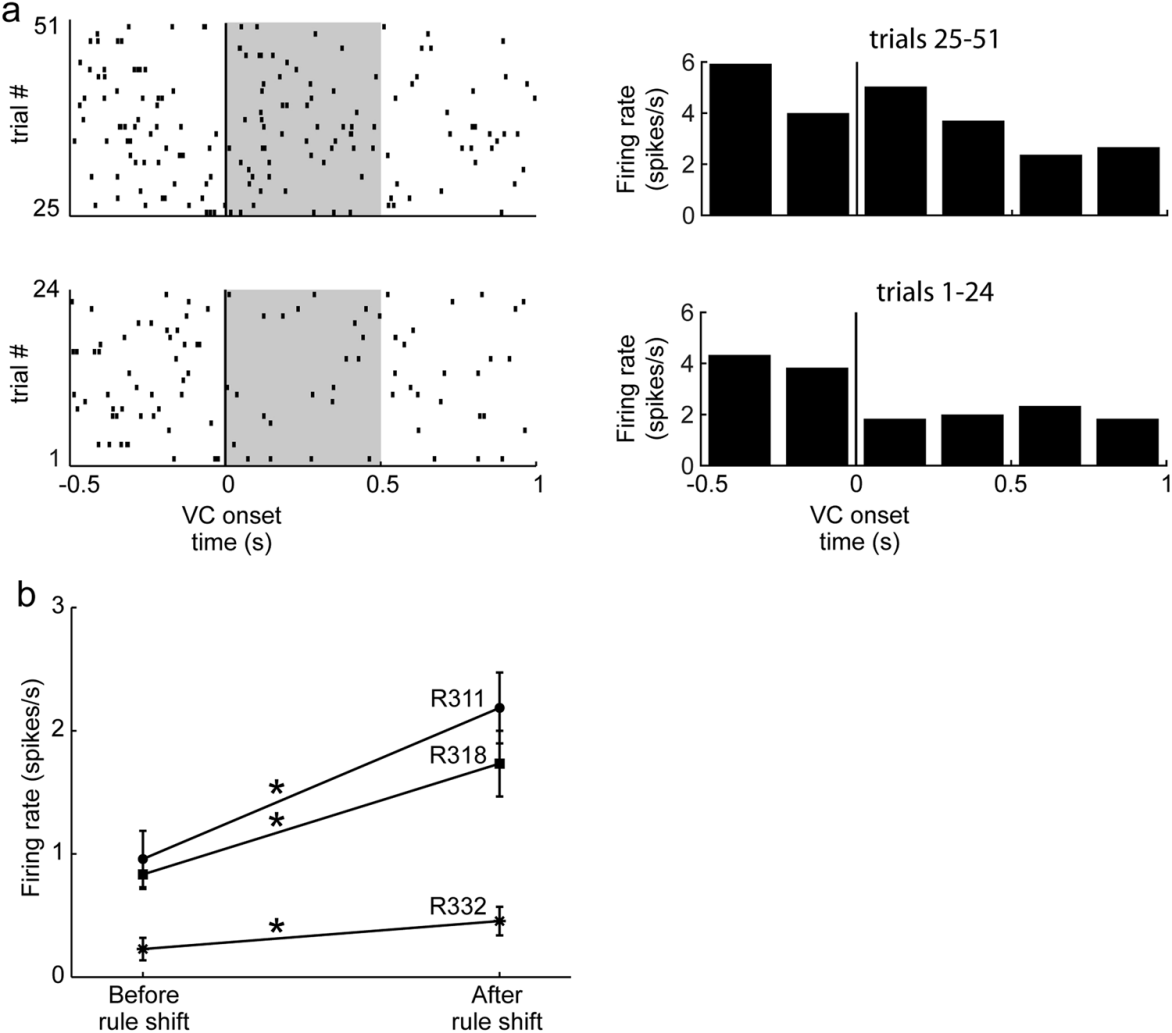
LC response to VC onset before and after first response contingency changes. (**a**) Example rasters of LC unit activity as a function of VC onset before (bottom, trials 1–24) and after (top, trials 25–51) the first contingencency changes from VC to the Turn rule. Corresponding PETHs are shown to the right of each raster display. (**b**) Mean and SEM of firing rate of the shaded area in (**a**) of LC units in three individual rats before and after the first rule shift. *p < 0.005 Wilcoxon test.

In all three rats pre-trained in the VC task, LC firing in the 0.5 s period *after* VC onset significantly increased in the trials after the first time the rats experienced a shift in response contingency (p = 5.9 × 10^−265^ for R328, 0.005 for R318 and 0.002 for R311, Wilcoxon rank sum test comparing numbers of spikes in the shaded 0.5 s windows in Fig. 3a prior to and after the rule shift; Fig. 3b). The increase in LC firing occurred while the animal continued adhering to the previous (VC) rule. This LC response elicited by the visual stimulus after the rule change was seen only in the first session involving response contingency change. This was the only situation where the LC firing increased after VC onset among all of the recordings.

### Do task-related responses change over the course of a session?

We examined the evolution of firing rates in the 1 s intervals prior to the three respective photodetector crossing events over the course of the recording sessions (0.5 s intervals for the reward photodetector, Rwd PD). A new block of trials was established after each rule change or strategy change. Pairwise comparisons generally showed one block with a firing rate significantly higher or lower than several other blocks in the same session (t-tests p < 0.05 with Holm-Bonferroni corrections; degrees of freedom varied among sessions, data not shown). However, firing rates did not consistently increase or decrease in comparisons between blocks with any particular strategies or rules (not shown). But in 12 of the 13 cases permitting this analysis, the earliest blocks showed higher firing rates than later ones (t-tests p < 0.05 with Holm-Bonferroni corrections, as above).

### Phasic changes in LC cell activity associated with task-related behaviors

**Figure 4:**
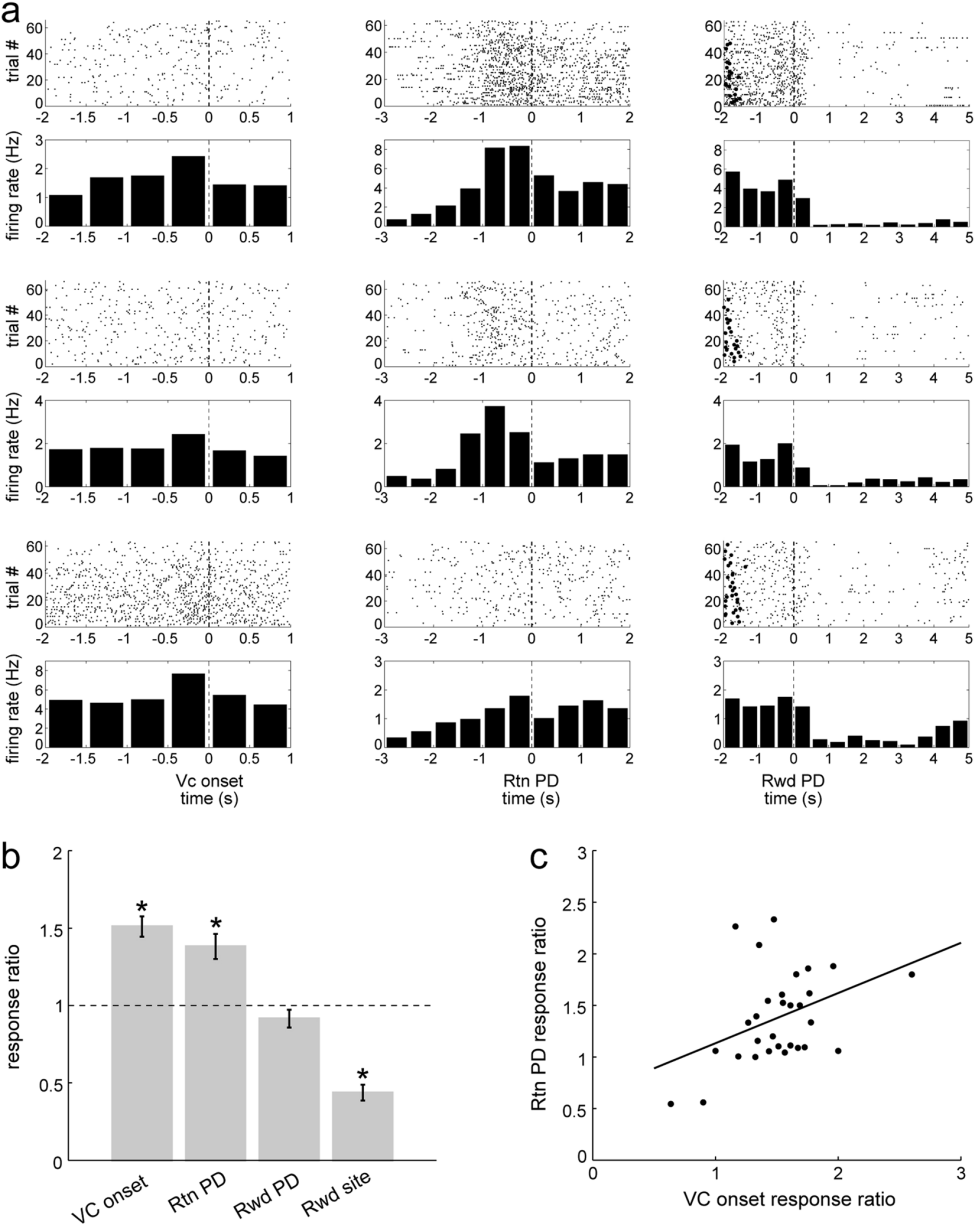
(**a**) LC activity around task events. Each row shows activity from a different recording. The third row is from an optogenetically identified noradrenergic neuron (rat R328). Left column, increased firing in the 500 ms before crossing the photodetector on the central arm, triggering VC onset. Middle column, activity increases prior to return arm (Rtn) PD crossing, triggering VC OFF. Right column, baseline activity prior to reward arm photodetector crossing (Rwd PD), followed by reduced activity at the reward sites. Black circles to the left indicate previous VC onset. Dashed vertical lines at 0 indicates the PD crossing for the respective task events for these trials. (**b**) Response ratios of the task events. A response ratio of 1 means no change, and greater (or less) than 1 indicates an increase (or decrease) in firing relative to baseline. Error bars show SEM. *Significant differences from 1 (see text). (**c**) Significant correlation between Rtn and VC onset response ratios for each recording (n = 30, r_(29)_ = 0.4, p = 0.03); VC onset responses tended to be greater than Rtn responses for individual neurons.

LC neurons showed several task-related changes in activity during performance on the T-maze (Fig. 4a). First, the LC neurons consistently increased firing at the beginning of each trial just prior to when the rat crossed the central arm photodetector, triggering VC onset (Fig. 4a, left column). To assess the magnitude of these changes, a response ratio was computed by dividing the mean firing rates of peak responses by the baseline activity rate in the window [−1.5 s, −1 s] preceding VC onset. This baseline was also used for all other response ratio calculations below. The peak response for VC onset was considered as the greater of the mean firing rates of the two bins preceding VC onset [−1 s, −0.5 s] and [−0.5 s, 0 s]. The VC onset response ratio exceeded 1.0 in 27 out of 30 sessions. The mean was 1.51 ± 0.07 (SEM, n = 30 sessions); this is significantly greater than 1 (one sample t-test, t_(29)_ = 7.7, p = 1.6 × 10^−8^; Fig. 4b).

LC activity also increased prior to the rat crossing the return arm (Rtn) photodetector, which triggered the VC off (referred to as “VC OFF”; Fig. 4a, middle column). After this, the firing rate returned to baseline levels. For these responses, the peak response was calculated the same way as for VC onset response. The response ratio exceeded 1.0 in 27 out of 30 sessions. The mean response ratio was 1.38 ± 0.08 (n = 30 sessions), significantly greater than 1 (one sample t-test, t_(29)_ = 4.7, p = 5.5 × 10^−5^; Fig. 4b).

To determine if the excitatory responses prior to VC onset and VC OFF were related, these response ratios were compared (Fig. 4c). The linear regression was significant (r = 0.4; df(29) p = 0.03), and the slope indicates that VC onset response ratios tended to be greater than VC OFF response ratios for individual neurons.

To avoid a possible confound with VC onset related activity, the response ratio for the reward arm (Rwd) photodetector crossings (Fig. 4a, right column) was calculated by dividing the mean activity in a briefer window only [−0.5 s, 0 s] before reward arm photodetector crossings by the baseline mean rate. The response ratio exceeded 1.0 in only 11 of 30 sessions. The mean ratio was 0.92 ± 0.06 (n = 30 sessions; Fig. 4b), not significantly different from 1 (one sample t-test, t_(29)_ = −1.5, p = 0.15). Thus, in contrast with VC onset and VC OFF, this photodetector crossing was not associated with an increase in firing rate even though it frequently triggered the (reward delivery) solenoid click.

As previously reported, LC activity decreased when the rat was at reward sites (Fig. 4a, right column), which could be related to reward consumption and/or a relatively low level of locomotor activity^6,10^. To quantify this decrease in activity, the response ratio was calculated as the mean activity in the interval [1 s, 2 s] after the reward arm photodetector crossings, corresponding to when rats reached the reward zone (confirmed from video records, not shown here) divided by the baseline firing rate. The response ratios were less than 1.0 in 28 out of 30 sessions. The mean of these reward response ratios was 0.44 ± 0.05 (n = 30), and values range from 0.08 to 1.10, significantly less than 1 (one sample t-test, t_(29)_ = 11.0, p = 7.2 × 10^−12^; Fig. 4b). The respective response ratios are compared in Fig. 4b.

To test if activity prior to PD crossing was a learned response, a control experiment was performed in one rat (R332) where LC activity was recorded during the initial acquisition of the VC task. During this training, LC activity was, surprisingly, highest during the background period ([−2, −1]s before VC onset PD crossing), but did not show the activity increases found in trained rats in Fig. 3 (not shown). This observation was replicated in three other VC training sessions with this rat. However, LC firing increased again within 0–0.5 s after the rat crossed the Rwd PD (paired t-test, t_(52)_ = −3.2, p = 0.002, not shown), before reaching reward sites.

### Correlations between LC firing rate and acceleration around task events

**Figure 5:**
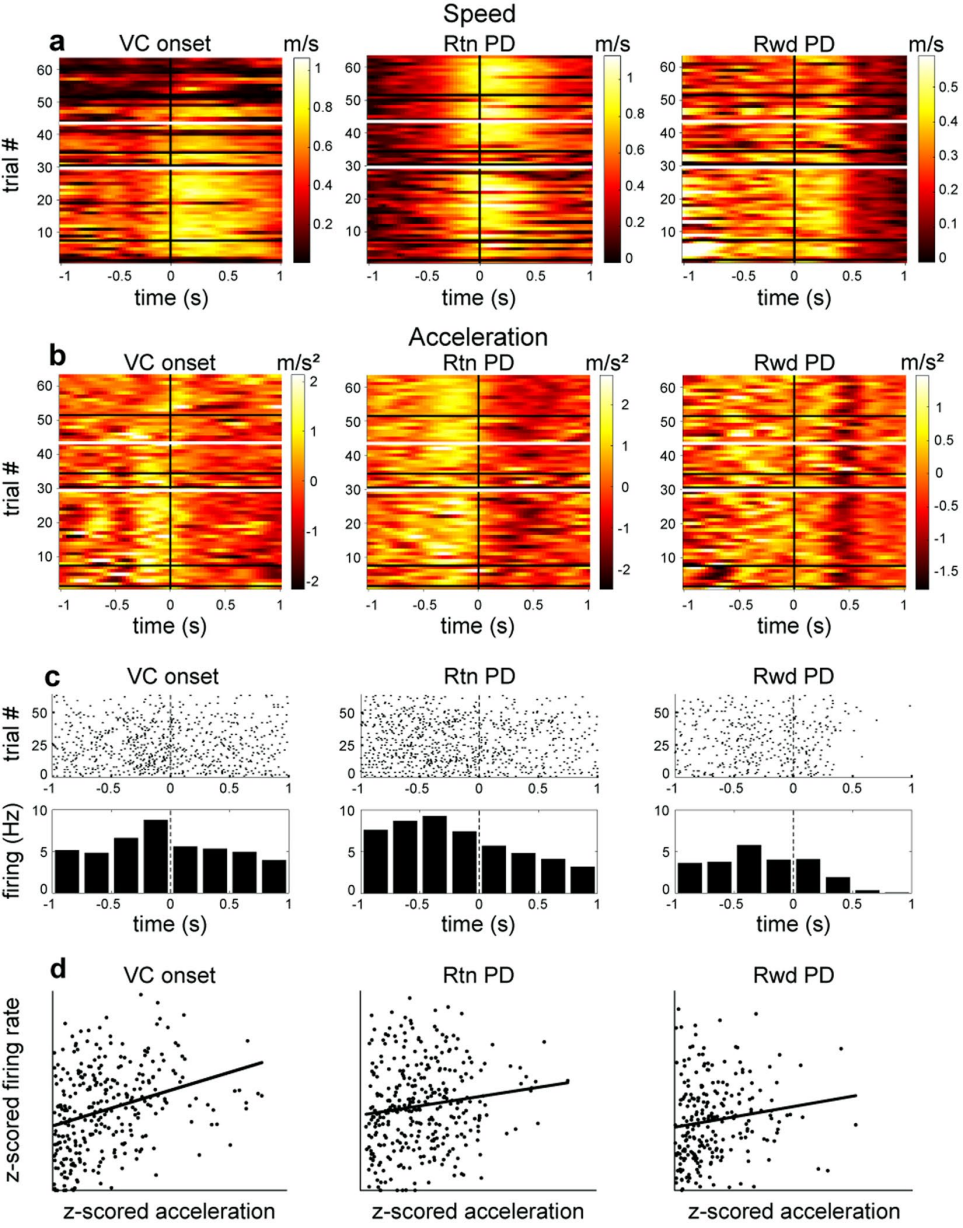
Relation between LC firing rate and acceleration around maze photodetector events. (**a**) In this typical session, speed increased in the 0.5 s period after photodetector crossings. White horizontal lines indicate when a rule shift was imposed and black horizontal lines indicate when the rat changed its behavioral strategy. (**b**) Acceleration increased in the 1 s prior to the respective photodetector crossings in the same session. (**c**) Corresponding raster and PETH plots of LC firing around the three task events. (**d**) LC firing rate correlation with acceleration around task photodetector events. The p values are 4.83E-07 for VC onset, 0.0268 for the Rtn photodetector, and 0.0324 for the Rwd photodetector.

Since the increase in neuronal activity occurred prior to photodetector crossings, we more closely examined the relation between LC activity and movement. The rats’ speed and acceleration were measured around the three principal task events (i.e., the photodetector crossings, Fig. 5a,b). Visual comparison of the color plots of acceleration (Fig. 5b) with peri-event time histograms (PETH) and raster plots of LC firing (Fig. 5c) suggests that both acceleration and LC firing rate increased during the same period in all rats, that is, during 0.5 s to 1 s before photodetector crossings. (In contrast, speed tended to increase after photodetector crossings, later than the onset of LC firing rate increases, and thus speed was not further considered as a possible correlate.)

Locomotor acceleration and deceleration can reflect different behavioral motivations and contexts. Because of the interest here in mobilization of effort, analyses only concerned positive values of acceleration (since negative values correspond to deceleration). Indeed, linear regression analyses confirmed a consistent positive correlation relation between firing rate and acceleration, for periods around the photodetector crossings. Figure 5d illustrates an example taken from one session. To confirm this observation at a population level, the firing rate and acceleration were examined from all recordings from each rat. Sampling bins (duration 200 ms) with smoothed firing rates less than 0.1 Hz were excluded. Data were z-transformed to render distributions more Gaussian and allow combination of data from neurons with different firing rates for each rat. In each rat, the data from all sessions combined showed highly significant correlations between firing rate and acceleration for data from each of the three photodetector crossing events, considered separately. See Table 1.

**Table 1:** p (and n) values of linear regressions of firing rate vs. acceleration of all session data  for each rat.

	VC Onset	VC OFF	Reward PD	Outside task events
Rat 311	7.45E-10 (3159)	2,30E-12 (1954)	5.56E-07 (3708)	3.16E-13 (3436)
Rat 318	4.75E-15 (2990)	6.31E-11 (1965)	8.97E-04 (3304)	2.58E-26 (3151)
Rat 328	1.88E-05 (2763)	3.69E-06 (1794)	2.81E-02 (2400)	2.00E-06 (2584)

In the control rat (R332) which had not been pre-trained and was recorded from the start of VC training, LC firing and acceleration were significantly correlated in only one of six sessions each in analyses of the VC ON and Rwd PD period (p values [with n’s in brackets] 0.057 [195], 0.15 [133], 0.84 [119], *0*.*02* [114], 0.83 [294], 0.96 [83] for VC ON and *0*.*02* [179], 0.92 [126], 0.86 [115], 0.53 [100], 0.99 [241], 0.29 [61] for Rwd PD). Accelerations developed in the same time windows around photodetector crossings as observed above in the well-trained animals, although they were lower in this animal, still in early stages of training.

### Are firing rate and acceleration correlated outside of task events?

Since LC activity increases were correlated with acceleration only in rats trained in the VC task, we tested whether these correlations were restricted to the three task events analyzed. Data were thus selected from time periods excluding the ±1 s time windows around VC onset and VC OFF as well as excluding the ±0.5 s around Reward photodetector crossings. Firing data were Gaussian smoothed and binned in 0.1 s periods and bins with firing rates less than 0.1 Hz (no spikes) were excluded to avoid excessive bias from reward and rest periods where acceleration and firing rate were low or zero. Before linear regression analysis, data were randomly down-sampled to match the sample sizes of the above analyses for task events. In the data grouped by animal for all sessions there was a statistically significant correlation between firing rate and acceleration for behaviors outside of task events (Table 1).

### Timing of LC firing onset relative to acceleration onsets

Two analyses examined the temporal relation between LC firing rate and acceleration. First, a sliding window method examined the period spanning ±1 s around the respective photodetector crossing events (see Methods section). Then acceleration data were shifted within each window in 20 ms steps. The linear correlation was computed for each step, based on the premise that the time shift yielding the greatest value of the correlation coefficient r would correspond to the best description of the temporal relationship between firing and acceleration. The analysis was repeated for each session and photodetector crossing event. Local maxima appeared for certain time shifts (not shown). The mean values were 90 ms for VC onset (n = 25), 34.5 ms for the return arm photodetector (n = 21), and 18.2 ms for the reward photodetector (n = 21). Note that the values are all positive indicating that for all three events, especially VC onset and return arm photodetector, LC firing preceded acceleration.

**Figure 6:**
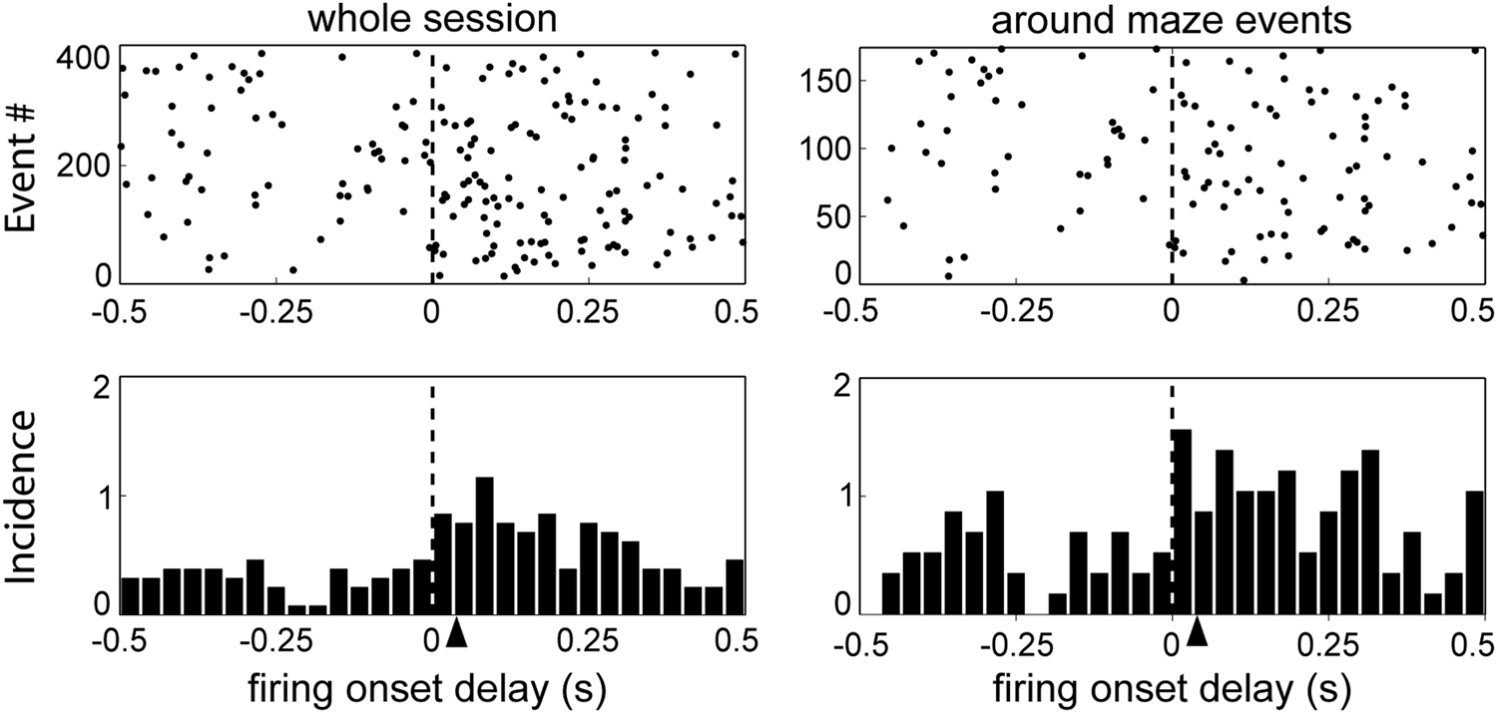
Cross-correlation acceleration onset relative to onset of LC firing increases during a representative session. The arrowheads indicate the mean lags of acceleration after firing onset.

The previous analysis examined all spike activity around the photodetector crossing events and thus suffered from the possible confound that since accelerations and the associated LC activity lasted over 0.5 s, spikes in each trial could have fired after the initial onset of the acceleration, even if the first ones may have preceded it. To deal with this issue, a second analysis compared the relative timing of the onsets of increases in firing rate and onsets of acceleration (see Methods). Since there were considerably fewer events than in the previous analysis, all task events were grouped together. The firing onsets were then used as trigger events for raster plots and PETHs of acceleration onset events within a ±500 ms window and this was repeated separately for all firing onset events for periods ±1 s around the three photodetector crossing task events and also for the entire session. Figure 6 shows the timing of acceleration onsets relative to LC firing increase for a representative session. Consistent with the previous analysis, the mean acceleration increase onsets occur after the firing rate increase onsets. For the 18 sessions with sufficiently high firing rates (see Methods section), the mean delay from LC firing increase onset to acceleration onset was 35.5 ± 4.3 ms for the whole session and 34.6 ± 5.1 ms around task events (Fig. 6).

## Discussion

This study is the first to document the responses of optogenetically identified noradrenergic LC neurons during unrestrained locomotion, permitting behavioral correlates of these identified units, including locomotor acceleration, to be observed. This complements other optogenetic studies of noradrenergic LC activity in set-shifting^18^, stress^19^ and novelty detection^20^ paradigms.

LC single and multiple units showed several task related activities in the T maze. First, in all sessions where the response-reward contingency was shifted for the first time, the onset of the visual cue elicited a response in LC. These responses were only seen in the very first task contingency shift, and this was the only condition when there was a response to the visual cue, itself. It is striking that this LC response to the VC occurred during the first trials where the VC was no longer relevant and the animal was required to shift attention and behavioral strategy, but was not seen when the VC was relevant for maintaining accurate behavioral performance. This sensitivity to novelty or environmental change, with rapid habituation, is a hallmark of LC neuron responses, as demonstrated in formal conditioning protocols^8,9^, spontaneous exploratory behavior^21^ or repeated exposure to sensory stimulation^7^. Note, however, that Sara and Segal^8^ observed LC responses at the beginning of extinction or upon reversal, i.e., when there was a reward prediction error. In contrast, here LC activation was not consistently associated with every change in reward contingency and behavioral strategy (see also refs^9,10^). The observation that LC neurons responded following VC onset after the rat was exposed to a new rule the very first time lends support to the proposal of Yu & Dayan^22^ that the LC-NE phasic activity signals ‘unexpected uncertainty’. The absence of LC activation upon later rule shifts could reflect habituation to reward contingency changes^7^.

The observation that task-related LC activity was greater in earlier trials than later ones of most sessions is consistent with earlier reports that LC neurons respond to conditioned stimuli early in a behavioral session but tend to disengage during overtraining^8^. Overall these results support a role for LC noradrenergic neurons in responding to changes in environmental contingencies^13^.

The second major, but unexpected, behavioral correlate of LC neurons found here was a consistent increase in firing rate just *prior* to photodetector crossings that triggered cue lights to go on or off. LC activity increases were reliably correlated with accelerations, but not speed. Increase in LC activity systematically preceded acceleration onset by about 35 ms. Sara and Segal^8^ found that LC neurons respond persistently to a preparatory signal that initiates the trial. Here, in the absence of such a signal, the rat’s ‘decision to go’ is associated with LC activation. Indeed, there is increasing evidence for the relation between the ‘go response’ and LC activation from diverse behavioral protocols in both rats and monkeys^13,23–27^. The present results showing LC activation at trial initiation are consistent with these reports.

This response to maze events triggered autonomously by the rat, was seen only in well-trained animals. Recordings from an untrained rat showed that acquisition of the task was required for these photodetector-crossing related behavioral correlates to be manifested. However, in this untrained rat, cells fired *after* reward photodetector crossings, when the reward delivery solenoid click sound would occur. LC neurons have been shown to respond to unexpected sensory stimuli. But responses to VC onset and VC OFF stimuli were not observed (perhaps because they were predictable), except on the first introduction of an extradimensional shift.

The delay between LC activation and acceleration onset is too brief for the LC activation to actually trigger the acceleration since it takes 50–100 ms for LC impulses to reach cortical targets (in non-human primates^28^) and it takes 100 ms for motor cortex to produce muscle contraction through activation of spinal cord^29^. Another possible pathway for LC to trigger movement would be through the superior colliculus where trajectory-selective responses appear, albeit earlier than in LC, several hundreds of ms before movement initiation^30,31^. Thus, both LC engagement and acceleration increases are more likely associated with mobilizing resources and systemic arousal^4^. This is consistent with LC receiving abundant input from motor-related nuclei in the midbrain, pons, medulla, and cerebellum, as revealed in a recent viral-genetic tracing study^32^.

Accelerated movement can reflect an increased motivational state and/or autonomic arousal as the animal initiates the trial or the effort to return to the start point to initiate the next trial. Note however that the VC OFF photodetector is located halfway down the return arm. Thus the animals had already initiated movement from the reward site when they accelerated and crossed it (not shown). An alternative interpretation is that the rats incidentally learned an instrumental association (that we unintentionally) embedded in the task. This would entail learning that movements at the photodetectors (which were visible, although the infrared photobeams were not) triggered light cues going on or off and the rats spontaneously learned to accelerate at these points, even though it did not directly change reward amounts or frequency. Triggering the cue onset or offset may have been sufficiently rewarding. If so, this then permitted the acceleration correlate of LC firing to be revealed serendipitously. Indeed, this study is the first to illustrate the responses of noradrenergic LC neurons to optical stimulation in rats with a wide range of unrestricted activity, so that behavioral correlates of these identified units, including locomotor acceleration, could be observed. Previous studies of task-related LC activity in behaving rats involved operant conditioning paradigms that did not require (or allow) much displacement of the animal within the experimental arena and there was no tracking of the rat’s instantaneous positions^8,10^. (And in monkeys, recording is always carried out with head restraint and movement restriction).

There is ample evidence that conditioned orienting responses occur in conjunction with goal directed motor responses^33–35^. The orienting response is associated with a number of physiological measures, including autonomic arousal, resulting in increased heart rate and respiration^36^, and these have been correlated with LC activity^24,37^. Thus, covariance of LC activity and acceleration is consistent with the proposal of Varazzani *et al*.^16^ that LC activation mobilizes energy for execution of an action and with an earlier proposal that LC activation, as part of the orienting response, provides a ‘cognitive complement’ to the sympathetic arousal^37^. Note that the VC onset and VC OFF photodetector crossing correlates were not observed in the recording of a rat not previously trained in the task. Furthermore, even though the rat did accelerate in these periods no significant correlations were found between firing rate and acceleration there. The previously trained rats would then have internally generated the orienting responses as they initiated the trial at the appropriate position on the maze in the absence of an explicit sensory cue signaling the beginning of the trial. This supports the view that the task related increase in LC firing before the photodetector crossing is part of a general conditioned orienting response, mobilizing resources, both physical and cognitive, to perform the trial. Overall these results support a role for LC noradrenergic neurons in mobilization of effort to face current challenges^4,16^.

## Materials and Methods

All experiments were carried out in accordance with local (Comité d’éthique en matière d’expérimentation animale no.59), institutional (Scientific Committee of the animal facilities of the Collège de France) and international (US National Institutes of Health guidelines; Declaration of Helsinki) standards, legal regulations (Certificat no. B751756), and European/national requirements (European Directive 2010/63/EU; French Ministère de l’Enseignement Supérieur et de la Recherche 2016061613071167) regarding the use and care of animals.

### Animals

Four male Long-Evans rats (Janvier Labs, Le Genest-Saint Isle, France; weight, 280–400 g) were maintained on a 12 h:12 h light-dark cycle (lights on at 7 A.M.). The rats were handled on each work day. To motivate animals for behavioral training on the T maze, food was restricted to 14 g of rat chow daily (the normal daily requirement) while water was partially restricted except for a 10–30 min period daily to maintain body weight at 85% of freely feeding weight. Rats were rehydrated during weekends.

### The automated T maze with return arms

The behavioral task (adapted from ref.^38^) took place in an area closed off by black curtains forming a large cylinder. Within this was an automated T maze elevated 70 cm above the floor (see Fig. 1a) consisting of a start area (width = 35 cm, length = 40 cm), a central arm (length = 100 cm), two reward arms (length = 50 cm each) and two return arms which connected the reward arms to the start area. Each arm was 8 cm wide, with 2 cm high borders. Small wells at the end of each reward arm delivered liquid reward (30 µl of 0.25% saccharin solution in water) via solenoid valves controlled by a CED Power1401 system (Cambridge Electronic Design, Cambridge, UK) with a custom-written script. Visual cues (VCs) were displayed on video monitors (76 cm above the ground, width = 88 cm, height = 53 cm) positioned behind, and parallel to the two reward arms, at a distance of 30 cm. The VCs were either lit or dim uniform fields. Photodetectors detected task events and triggered cues and rewards via the CED Spike2 script.

### Behavioral training and tasks

#### Pre-training

Rats were first habituated to the T maze for several days. The visual cues were switched off during pre-training. Droplets of water were scattered over the maze to encourage foraging in the partially water-deprived rats. Then the water droplets were restricted to the two reward wells. After rats learned the reward site locations, they were shaped to proceed in one direction on the maze, starting up the central arm, selecting a reward arm, consuming reward and then following either return arm to the start area without backtracking. Rewards were provided on both arms. When the rat had learned to follow the correct path, VC task training began.

#### VC task training

Room lights were dimmed. The rat was placed in the start area. The trial was initiated when the rat crossed the central arm photodetector triggering one of the two screens to light up in a pseudorandom sequence from trial to trial (programming of the sequence is described below). A correct response was scored when the rat entered the reward arm in front of the lit screen side regardless of whether it was on the left or right side (Fig. 1b, above). The photodetector in the middle of the reward arm triggered reward delivery with an audible solenoid click. Crossing photodetectors in the return arms triggered extinction of the VC. When a rat reached criterion performance in the VC task (8 consecutive correct choices for two successive sessions), VC training ended and the animal was prepared for electrode implantation surgery. Rats required 3–4 sessions (250–360 trials in total) to reach this criterion. Three rats were pre-trained in the VC task, including one which had been injected with the virus for optogenetic stimulation of LC (rat R328). A fourth animal (R332) was not pre-trained in the VC task as a control, and analyses and results below do not include this rat, unless indicated.

#### The visual and place discrimination tasks

One week after the surgery, rats were recorded as they alternated between visual and spatial discrimination reward contingency rules. There was no signal indicating that the rule had been changed; the rat could only detect this by the change in response-reward contingencies. Under the latter ‘Turn’ (T) rule, rewards were only provided on one pre-chosen reward arm (independent of which side the VC appeared on, Fig. 1b, bottom). The rewarded arm was selected as the non-preferred side for that rat, as determined during pre-training. Rule changes were imposed after the rats demonstrated their attention to the appropriate cues by achieving criterion performance. The protocol is referred to as extra-dimensional shift (EDS) since the two reponse-reinforcement contingencies require shifting attention between different sensory modalities.

The first EDS session always began with the VC rule. Subsequent shift sessions started with the rule that the rat had been following at the end of the previous session. The rule was switched when the rats reached a criterion performance of at least 18 correct out of the last 20 trials, computed with a moving window. This stringent criterion was used to achieve a relatively stable long baseline recording. This also assured that the relevant cues dominated the attentional set of the rat.

#### Programming the pseudorandom sequence

In both the VC and the T tasks, the sequence of left/right illumination of screens was programmed according to a pseudorandom sequence to avoid possible confounds among possible strategies in the following manner:

1. the screen on a given side was never lit on more than 2 consecutive trials;
2. left/right alternations of the lit screen did not repeat more than twice;
3. in any eight successive trials, there were no more than five trials with the screen lit on the same side.

### Viral vector preparation

The Canine Adenoviral vector (CAV2-PRS-ChR2-mCherry) was produced at the University of Bristol using previously described methods^39^. This CAV2 viral vector expresses channelrhopsin-2 (ChR2) under the control of PRSx8 (synthetic dopamine beta hydroxylase promoter) which restricts the expression of the transgene to noradrenergic (NA) neurons (Figure VI.1a in ref.^40^; also^41^). The titer, assayed by transducing DK cells and then by immune-staining for expression of viral hexon protein, is 1.2 × 10^12^ transducing units/ml. Aliquots of virus were stored at −80 °C before stereotaxic injection. The viral vector was diluted 5x in sterile phosphate-buffered saline (PBS) before use. The specificity of this vector was confirmed previously *in vitro* and *in vivo*^39^. The specificity of the responses was further supported by the observations of no excitatory responses to laser stimulations in histologically verified LC recordings in a rat with poor transduction of the LC after an off-target CAV injection (not shown).

### Virus injection

In one rat (R328), 4 months before the electrode implant surgery, CAV2-PRS-ChR2-mCherry was injected into the right LC. The rat was anesthetized with sodium pentobarbital (40 mg⁄kg, with 5 mg sodium pentobarbital as a supplement every hour) intraperitoneally. Body temperature was kept constant at 37° with a heating pad (Harvard Apparatus Limited). The scalp was shaved and disinfected. The head was fixed to a stereotaxic apparatus with dull ear bars with bregma and lambda in the same horizontal plane. The eyes were covered with thick gauze moistened with 0.9% NaCl. The site corresponding to LC position was marked on the exposed skull for injection in right LC (AP ~3.9 mm relative to lambda, ML ~1.2 mm) and a trephine hole was made (~2 mm diameter). A micropipette (calibrated in 1 µl intervals, Corning Pyrex) with a tip diameter of 20 µm was connected to a Hamilton syringe, and backfilled with 1 µl of the diluted viral vectors. Microinjections of 0.33 µl were made into the LC (AP −3.8~−4 mm relative to lambda, ML 1.1~1.2 mm, with a 15° rostral tilt) at three sites dorsoventrally (5.2, 5.5, 5.7 mm below the brain surface). The pipette was left at each depth for an additional 3–5 min before moving down to the next site. When the injection was finished, the trephine hole was covered with sterilized wax and the scalp was sutured. The rat was observed until recovery and was then singly housed.

### Electrode and optrode implants

Following VC task pre-training, at least one day before surgery, rats were returned to *ad libitum* water and food. General surgical preparation is described in the previous section. Moveable tungsten microelectrodes (insulated with epoxylite, impedance = 2–4 MΩ, FHC Inc, USA) were used for LC recordings. A single microelectrode, or two or three such electrodes glued together was implanted at AP −3.8~−4 mm relative to lambda, and ML 1.1 ~ 1.2 mm, with a 15° rostral tilt. A stainless steel wire (Teflon coated, diameter = 178 µm, A-M systems Inc), implanted in the midbrain area, about 1–2 mm anterior to the LC electrode tip, served as a fixed LC reference electrode, permitting differential recording. The rat with the virus injection (R328) was implanted with an optrode made of a tungsten microelectrode (insulated with epoxylite, impedance = 2–4 MΩ, FHC Inc, USA) glued to a 200 µm optic fiber implant with a ferrule (0.37 numerical aperture, hard polymer clad, silica core, multimode, Thorlabs), with tip distances 1 mm apart (the electrode was deeper). The optic fiber implant and optic fiber cables were constructed at the NeuroFabLab (CPN, Ste. Anne Hospital, Paris. Two screws (diameter = 1 mm, Phymep, Paris) with wire leads were placed in the skull above the cerebellum to serve as ground. LC electrodes were progressively lowered under electrophysiological control until characteristic LC spikes were identified (located ~ 5–6 mm below the cerebellar surface, see ref.^10^ for details). For the virus-injected rat, LC spikes could also be identified by responses to laser stimulations (described below). Following implantation, the microelectrode was fixed to a micro-drive allowing for adjustments along the dorsal-ventral axis. The headstage was fixed to the skull with dental cement and surrounded by wire mesh for protection and shielding. After the surgery, animals were returned to their home cages for at least one-week recovery with ad libitum water and food and regular observation.

### Electrophysiological recordings

Rats were then returned to dietary restriction. The movable electrodes were gradually advanced until a well discriminated LC unit was encountered and then all channels were recorded simultaneously while the rat performed in the T maze. If no cells could be discriminated, the electrodes were advanced and there was at least a 2 h delay before the next recording session.

For daily online monitoring of LC spikes, pre-amplified signals were filtered between 300–3000 Hz for verification on the computer screen (Lynx-8, Neuralynx, Bozeman, MT, USA) and also transmitted to an audio monitor (audio analyzer, FHC). For recordings, brain signals were pre-amplified at unity gain (Preamp32, Noted Bt, Pecs, Hungary) and then led through a flexible cable to amplifiers (x500, Lynx-8, Neuralynx) and filters (0.1–9 kHz, Lynx-8, Neuralynx). Brain signals were digitized at ~20 kHz using CED Power1401 converter and Spike2 data acquisition software. The LC unit activity was identified by: 1) spike waveform durations ≥0.6 ms; 2) low average firing rate (1–2 Hz) during quiet immobility; 3) brief responses to unexpected acoustic stimuli followed by prolonged (around 1 s) inhibition; 4) for the virus-injected rat (R328), LC units were verified by responses to laser stimulation. A laser driver (Laserglow Technologies, Canada, wavelength 473 nm) was controlled by signals from a stimulator (Grass Technologies, USA, Model SD9). Light intensity from the tip of optic fiber was measured by a power meter (Thorlabs, Germany, Model PM100D). In pilot acute experiments we determined the parameters for laser stimulation to be 2 Hz, 100 ms pulse duration (Hickey *et al*., 2014, Li *et al*., 2016) with light intensity kept at 10 mW. If unit firing was entrained to the pulses with an increased rate (to at least twice the baseline firing rate) averaged over all the stimulations, it was considered to be a noradrenergic LC unit.

A light emitting diode (LED) was mounted on the cable that was plugged into the headstage. This was detected by a video camera mounted above the T-maze and transmitted to the data acquisition system at a sampling rate of ~30 Hz.

### Tissue processing

After all recording experiments, electrolytic lesions (40 µA, 10 s cathodal current) were made at the tip of the electrodes. Rats were then injected with a lethal dose of sodium pentobarbital and perfused transcardially with 0.9% NaCl followed by 10% formalin solution. The brains were removed and post-fixed overnight in the same solution at 4 °C and were cryo-protected by immersion firstly in 20% sucrose (dissolved in 0.1 M phosphate buffer (PB) for 48 h then in 30% sucrose (dissolved in 0.1 M PB) for an additional 48 h  Brain slices were cut coronally at a thickness of 40 µm with a freezing microtome and were collected in cold 0.1 M PB for subsequent  Nissl staining. Recording site positions were reconstructed by interpolation from the final position of the lesion on the basis of records of the electrode descent. Recordings at sites with reconstructed electrode positions outside LC proper were excluded from analysis.

### Fluorescent immunohistochemistry

Sections were cut on a freezing microtome (40 µm thickness) and were rinsed 3 times (5 min each) in PBS, then permeabilized with 3 washes (5 min each) in PBS with 0.2% Triton X-100 (Sigma-Aldrich). Sections were then quenched with 0.1 M glycine for 20 min. After that, they were placed in 10% normal goat serum (NGS, Thermoscientific) in PBS at room temperature (RT) for 1 h to block nonspecific antigens. Sections were then incubated in primary antibodies overnight at 4 °C in darkness with both chicken anti-tyrosine hydroxylase (TH) antibody (1:500, Abcam) and mouse anti-mCherry antibody (1:200, Ozyme) in PBS containing 0.1% Triton X-100 and 3% NGS. After three 5 min rinses in PBS, sections were then incubated with secondary antibodies in PBS containing 3% NGS for 1 h at RT in darkness. Secondary antibodies used in this study were Alexa Fluor 488 goat anti-chicken IgG (1:3000, Life Technologies) and Alexa Fluor 546 goat anti-mouse IgG (1:3000, Life Technologies). Then slices were washed three times (5 min each) in PBS, mounted onto slides (Superfrost) and coverslipped with fluorescence mounting medium (DAKO).

### Signal processing and spike sorting

For off-line spike detection of LC activity in three rats, the wide-band signals were converted and digitally high-pass filtered (nonlinear median-based filter). Waveforms with amplitudes passing a threshold were extracted and then subjected to principal component analysis (PCA). All of these processes were performed with NDManager^42^ (http://neurosuite.sourceforge.net). Spikes were sorted with a semi-automatic cluster cutting procedure combining KlustaKwik (KD Harris, http://klustakwik.sourceforge.net) and Klusters (L Hazan, http://neurosuite.sourceforge.net). Spikes with durations less than 0.6 ms were rejected. In one rat (R311) the LC signal was filtered from 300–3000 Hz during recording, and the spike sorting was performed with Spike2 software (which employs a waveform template matching algorithm). All data analyses were performed using Matlab (R2010a) with the statistical toolbox FMAToolbox (developed by M. Zugaro, http://fmatoolbox.sourceforge.net).

### Experimental design and statistical analysis

For each unit, a stable baseline period outside of task events was selected for comparison to activity around task events. In cases of normally distributed data, parametric statistics are employed. Paired t-tests compared the LC spike counts for the same unit relative to a baseline period in the same trial. Data are presented as means ± standard error of the mean (SEM) for individual sessions. Numbers of observations are indicated in the text or figure legends. A one-sample Student’s t-test was used to compare the response ratios with the null hypothesis value of 1. For comparisons of LC spike counts between trial blocks before vs. after rule shifts during the three first rule-shift sessions, the Wilcoxon rank sum test was applied. T-tests were used to compare the spike counts between trial blocks with different rules or strategies. For all statistics the criterion for significance is p < 0.05, unless otherwise noted.

### Regression analyses of firing and acceleration

The firing rate of LC activity of each trial was calculated in 200 ms bins within the time window of ±1 s around each maze event including VC onset, reward arm photodetector crossing and return arm photodetector crossing (VC OFF). Bins with firing rates less than 0.1 Hz were removed. The instantaneous speed was calculated as the distance traversed by the head-mounted LED divided by the video sampling time interval (33 ms). This was Gaussian smoothed (kernel size = twice the standard deviation of the Gaussian distribution). The instantaneous acceleration was then computed as the change of speed divided by the time interval. Acceleration was calculated as the mean of all of the instantaneous acceleration measures in each 200 ms bin. To permit comparisons among sessions, accelerations and firing rates were normalized by z-score transforms.

### Firing increase onset and acceleration increase onset

Instantaneous firing rate for each 50 ms bin was estimated with the same Gaussian kernel. The threshold for onset of firing rate increases was defined as the value delimiting one tail in the distribution of firing rates (95th percentile) over the session. The threshold for detection of the onset of acceleration increase was set at the 97.5th percentile (p < 0.025) to avoid false positives. Sessions with firing increase onset thresholds ≤4 Hz were excluded from analysis since a single spike can reach 4 Hz in this calculation of instantaneous firing. This analysis yielded time stamps of onsets of firing increases and of acceleration increases.
